# Neural predictors of cognitive improvement by multi-strategic memory training based on metamemory in older adults with subjective memory complaints

**DOI:** 10.1038/s41598-018-19390-2

**Published:** 2018-01-18

**Authors:** Soowon Park, Seung-Ho Ryu, Yongjoon Yoo, Jin-Ju Yang, Hunki Kwon, Jung-Hae Youn, Jong-Min Lee, Seong-Jin Cho, Jun-Young Lee

**Affiliations:** 1 Department of Education, Sejong University, Seoul, Republic of Korea; 2 Department of Psychiatry, School of Medicine, Konkuk University, Konkuk University Medical Center, Seoul, Republic of Korea; 3 Seoul National University College of Medicine, Seoul, Republic of Korea; 4 Department of Biomedical Engineering, Hanyang University, Seoul, South Korea; 5 Department of Neurology, Yale University School of Medicine, New Haven, CT USA; 6 Graduate School of Clinical Counseling Psychology, CHA University, Pocheon, Republic of Korea; 7 Department of Psychiatry, Gachon University Gil Medical Center, Incheon, Republic of Korea; 8 Department of Psychiatry and Neuroscience Research Institute, Seoul National University College of Medicine, Seoul, Republic of Korea; 9 Department of Psychiatry, SMG-SNU Boramae Medical Center, Seoul, Republic of Korea

## Abstract

Previous studies have indicated that memory training may help older people improve cognition. However, evidence regarding who will benefit from such memory trainings has not been fully discovered yet. Understanding the clinical and neural inter-individual differences for predicting cognitive improvement is important for maximizing the training efficacy of memory-training programs. The purpose of this study was to find the individual characteristics and brain morphological characteristics that predict cognitive improvement after a multi-strategic memory training based on metamemory concept. Among a total of 49 older adults, 39 participated in the memory-training program and 10 did not. All of them underwent brain MRIs at the entry of the training and received the neuropsychological tests twice, before and after the training. Stepwise regression analysis showed that lower years of education predicted cognitive improvement in the training group. In MRI, thinner cortices of precuneus, cuneus and posterior cingulate gyrus and higher white matter anisotropy of the splenium of corpus callosum predicted cognitive improvement in the training group. Old age, lower education level and individual differences in cortical thickness and white matter microstructure of the episodic memory network may predict outcomes following multi-strategic training.

## Introduction

Older adults are more likely to experience subjective cognitive impairment^1^ and such age-related cognitive impairment may induce many difficulties in their daily lives^2^. Many memory-training programs were developed to improve older adults’ memory abilities^3,4^, using different techniques ranging from a simple memory repetition to multi-strategic memory trainings such as imagery, organization or method of loci^5,6^. However, it is important to predict, among the elderly population, those who can benefit from such a memory training before the actual training. Many elders have achieved cognitive improvement through memory-training programs but still some others have not^7^. If researchers can predict which participants can acquire cognitive improvement before training, it could be helpful in deciding which cognitive training to use or not to.

In order to design an effective intervention to improve cognitive abilities, previous studies have explored the role of individual characteristics such as motivation (e.g., need for cognition, implicit theories of intelligence)^8^, demographic variables or cognitive abilities at the entry (e.g., age, education)^9^. These days, with the development of brain imaging techniques, growing attention has been placed on the differences in neural structure and network for predicting successful outcomes of memory training or therapy in healthy young adults (19–24 years old)^10^, adults with subjective memory complaint (42–77 years old)^11^ or patients with traumatic brain injury (25–65 years old)^12^. For instance, Engvig and colleagues (2012) have found that baseline hippocampal CA2/3-volume can predict the improvement of verbal memory after the implementation of method-of-loci mnemonic technique and everyday memory strategies^11^.

In the present study, we extend this line of research by gathering data from a new sample of Korean older adults with subjective memory complaint aged from 61 to 81 and by analyzing their demographics, initial cognitive abilities and whole brain structures as possible predictors of training outcome of Multi-strategic Memory Training (MMT), a multi-modal memory training program^13^. We explored the demographical, neuropsychological and brain morphological factors at baseline in order to predict the cognitive improvements.

Educational outcomes are determined by the combination of an individual’s prior traits and instructional strategies^14^. Especially, since the morphology of the brain is one of the most prominent biological structures that could determine the cognitive ability, it would be crucial to consider the brain structure as a variable for predicting training efficacy. Previous studies have tested the brain-cognition connection by providing evidence that individual variations in brain morphology can predict the performance difference of perceptual task^15^ and auditory task^16^ and the difference in learning performance in math tutoring^17^. These studies have led to the general consensus that an individual’s training outcome is related to the structural differences of the brain.

Investigating baseline brain structures closely related with future training outcomes may provide knowledge in the appropriate memory-training program for each participant. There is a large educational demand for training in older adults experiencing memory problems, and individual differences can predict the relevant intervention outcomes in healthy elder population. This study investigates which baseline individual characteristics or brain morphology can predict MMT^13^ outcomes in elder adults with subjective memory complaint. The MMT training is a multi-strategic training based on the metamemory concept and focused not just on the memory but also on the transfer effects on general cognitive functions.

Metamemory, or the self-awareness of memory, has not been consistently defined across studies because of its multifaceted characteristics. In this study, we follow the metamemory frameworks of Kasznizk and Zak (1996)^18^ that are derived from abundant psychological and neurological studies^19^. Metamemory encompasses knowledge of memory (e.g., formation of short-term memory, working memory, long-term memory), monitoring of one’s memory based on the knowledge of memory (e.g., monitoring of one’s memory process) and control of memory (e.g., selection of proper mnemonic strategies)^20^. This MMT program emphasizes learning about the cognitive processes of memory and multiple strategies of cognitive control and memory and having self-efficacy in learning and understanding people’s knowledge about their memory. The metamemory training program is similar to existing memory training programs in that the participants learn memory strategies, but discriminates itself from others in that it trains the participants the ability to monitor their memory performances based on the knowledge of the memory. Therefore, it is hypothesized that participants who have less experience in monitoring and controlling their memory performance would experience higher levels of improvement by the MMT.

Furthermore, based on the previous study that the baseline hippocampal CA2/3-volume can predict improvements in verbal memory^11^ and hippocampus is one of the most crucial regions for memory function^21^, we also investigated the hippocampal gyrus and related tracts as possible predictive regions. Since the MMT focused on the improvement of both general cognitive and memory functions, a large range of transmission and integration of operations in various brain areas such as frontal and hippocampal regions is needed. Therefore, we hypothesized that white matter fiber system that coordinates communication between frontal lobe and hippocampus is related to the MMT outcomes^22,23^.

However, insufficient and inconsistent research on the neural predictors for training outcomes hinders the formulation of any definitive hypothesis. Thus, we performed wholebrain voxelwise analyses to assess cerebral gray and white matter predictors of MMT. In the current study, stepwise multiple regression was used to investigate demographical and neuropsychological characteristics and cortical thickness and diffusion tensor imaging was used to discover the predictors of cognitive improvement.

## Results

### Baseline demographic and neuropsychological characteristics of the participants

Baseline demographic and neuropsychological characteristics of the participants are presented in Table 1. The mean age of the training group (*n* = 39) was 69.82 (*SD* = 4.90) ranging from 65 to 77; their mean years of education was 11.41 (*SD* = 4.31, minimum = 3, maximum = 25); and their average score of MMSE was 27.13 (*SD* = 2.67). The mean age of the control group (*n* = 10) was 70.40 (*SD* = 3.95) ranging from 61 to 81; their mean years of education was 13.00 (*SD* = 4.00, minimum = 3, maximum = 25); and their average score of MMSE was 27.30 (*SD* = 2.50). There were no differences in age, education, MMSE and gender distributions between the training and the control group.

**Table 1:** Demographic variables and MMSE scores for each group.

	Group		*t or χ* ^2^	*p*	Total (*N* = 49)
	Training (*n* = 39)	Control (*n* = 10)			
Age (years)	69.81 (4.90)^a^	70.40 (3.95)	0.35	0.731	69.94 (4.69)
Education (years)	11.41 (4.31)	13.00 (4.00)	1.01	0.297	11.73 (4.26)
Gender (M:F)	11:28	6:4	3.55	0.075	17:32
MMSE	27.13 (2.67)	27.30 (2.50)	0.18	0.855	27.16 (2.61)

^a^*M* (*SD*), M: Male, F: Female, MMSE: Mini-Mental State Examination.

### Neuropsychological characteristics of the training and control group

Results of tests that evaluate verbal memory (i.e., Elderly Verbal Learning Test) and nonverbal memory (i.e., Simple Rey Figure Test), executive function (i.e., Digit Span Test, Spatial Span Test) and language ability (i.e., Categorical Fluency Test, short version of Boston Naming Test; sBNT) pre- and post-training are presented in Table 2.

**Table 2:** Neuropsychological characteristics for the training group.

Measure	Training (*n* = 39)	
	Pre	Post
Verbal memory		
Immediate free recall^†^	28.97 (5.94)^a^	31.13 (6.7)
Delayed free recall	5.21 (2.32)	5.36 (2.8)
Visuospatial Memory		
SRFT copy	14.58 (1.89)	14.87 (1.5)
SRFT delayed recall	10.59 (3.63)	11.5 (3.63)
Attention		
DST forward	6.15 (1.16)	6.26 (1.09)
DST backward	4.28 (1.02)	4.49 (1.19)
VST forward	5.67 (0.96)	5.44 (0.91)
VST backward	5.36 (1.14)	5.44 (1.23)
Fluency		
Categorical fluency	28.67 (5.29)	30.18 (5.87)
Language		
Boston Naming Test	12.21 (2.39)	12.62 (1.98)

^a^*M* (*SD*), ^†^Summation of total numbers (out of 45) of 5 times immediate recall of the word list; ^††^*p* value from training group vs. control group-by-time interaction, SRFT: Simple Rey Figure Test, DST: Digit Span Test, VST: Visual Span Test.

### Changes in cognitive function

The standardized neuropsychological assessment scores (i.e., z-scores) pre- and post-training in the training group are presented in Supplementary Figure 1 (left). The same data from the control group are presented in Supplementary Figure 2 (left). The change in each cognitive ability was computed by subtracting pre-z-score from post-z-score, the results indicating the relative cognitive change. Median changes in standardized cognitive scores were 0.059 in the training group and −0.011 in the control group, respectively.

### Demographic and Neuropsychological Predictors

Stepwise multiple regression including all the baseline values of the scores of neuropsychological tests, age, gender and years of education revealed that in the training group, only the years of education (*β* = −0.381; *p* = 0.017) significantly predicted the performance change in cognitive function (adjusted *R*^2^ = 0.122; *F*(1, 37) = 6.269; *p* = 0.017) (Table 3). Participants with lower years of education experienced a larger increase in cognitive function after training. In the control group, the regression equation was not significant; in other words, nothing predicted cognitive change.

**Table 3:** Demographic and neuropsychological predictors for the training group.

Variables	Training group (*n* = 39)					
	*R* ^2^	*B*	*SE*	β	*t*	*p*
DV: Change in cognitive function						
IV: Education	0.122*	−0.30	0.012	−0.381	2.504	0.017

*p < 0.05, Regression equation was not significant in the control group. DV: dependent variable, IV: independent variable, *B* = unstandardized regression coefficient, β = standardized regression coefficient.

### Associations between brain morphology and cognitive change following MMT

#### Cortical thickness

**Figure 1:**
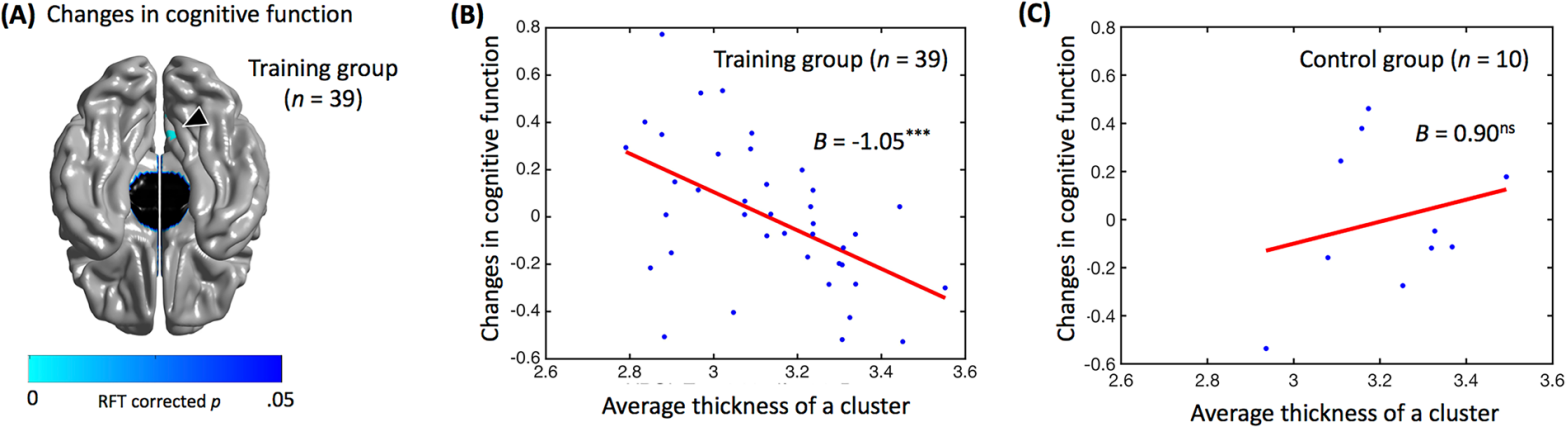
(**A**) Brain map showing the clusters with a significant correlation between cortical thickness and changes in cognitive function at right precuneus (BA 7), cuneus (BA 17) and cingulate gyrus (BA 31) in the training group, which emerged when the P map indicated by the color was corrected for multiple comparison at a 0.05 threshold. The cluster is indicated by the triangle. (**B**) Regression graph of the averaged cortical thickness of clusters at the entry of the training according to changes in cognitive function in the training group (adjusted *r*^2^ = 0.32; *F* = 5.52; *p* < 0.005; beta = −1.05; *t* = −4.22; *p* < 0.001). (**C**) Regression graph of the averaged cortical thickness of clusters at the entry of the study according to changes in cognitive function in the control group (adjusted *r*^2^ = 0.32; *F* = 0.45; *p* = 0.768; beta = 0.90; *t* = 0.94; *p* = 0.391). ***p < 0.001, *ns* = non-significant.

In the training group, significant relationships between the cortical thickness and changes in cognitive function [Random Field Theory (RFT) corrected; *p* < 0.05; overall model’s adjusted *r*^2^ = 0.32; *F* = 5.52; *p* < 0.005; cluster area = 237.47 mm^2^] were found in the area consisting of right precuneus (BA 7), cuneus (BA 17) and posterior cingulate gyrus (BA 31) [beta = −1.05; *t* = −4.22; *p* < 0.001; number of vertex = 231; peak MNI coordinate (x, y, z) = (4.90, −68.97, 29.38)]. Figure 1A indicates the brain regions showing significant correlations with the changes in cognitive function in the training group. The regression graph of the averaged cortical thickness on cluster at the entry of the training versus changes in cognitive function is presented on Fig. 1B. In the control group, there was no significant relationship between cortical thickness and changes in cognitive function (Fig. 1C).

#### White matter microstructure

**Figure 2:**
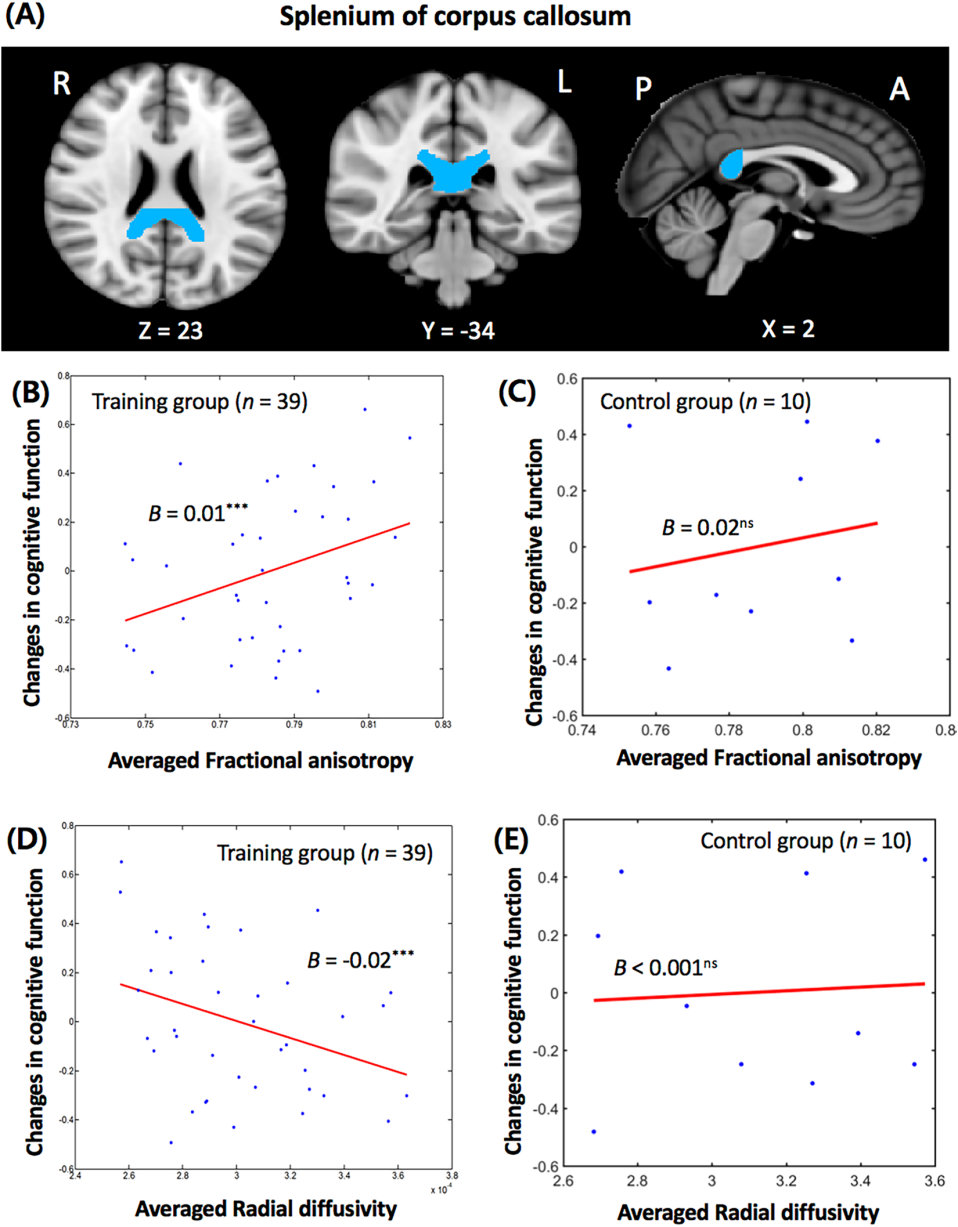
(**A**) The location of the splenium of corpus callosum (MNI coordinates: 23, −34, 2). (**B**) The relationship between the averaged fractional anisotropy and the changes in cognitive function (overall model’s adjusted *r*^2^ = 0.34; *F* = 6.22; *p* < 0.001; beta = 0.0051; *p* < 0.001) of the splenium of corpus callosum in the training group. The results were significant at a corrected false discovery rate-corrected *p* < 0.05 to control for the multiple comparisons. (**C**) The relationship between the averaged fractional anisotropy and the changes in cognitive function (overall model’s adjusted *r*^2^ = 0.06; *F* = 0.83; *p* = 0.52; beta = 0.018; *p = *0.47) of the splenium of corpus callosum in the control group. (**D**) The relationship between the averaged radial diffusivity and the changes in cognitive function (overall model’s adjusted *r*^2^ = 0.37; *F* = 7.04; *p* < 0.001; beta = −0.0193; *p* < 0.001) of the splenium of corpus callosum in the training group. The results were significant at a corrected false discovery rate-corrected *p* < 0.05 to control for the multiple comparisons. (**E**) The relationship between the averaged radial diffusivity and the changes in cognitive function (overall model’s adjusted *r*^2^ = 0.01; *F* = 0.96; *p* = 0.47; beta < 0.001; *p* = 0.82) of the splenium of corpus callosum in the control group.

In the training group, diffusion tensor imaging (DTI) analysis showed that the changes in cognitive function were positively correlated with the averaged fractional anisotropy (FA) values and negatively correlated with the averaged radial diffusivity (RD) values in the right splenium of corpus callosum after controlling for age and gender (Fig. 2B,D). For the FA analysis, the overall model’s adjusted *r*^2^ = 0.34, *F* = 6.22, *p* < 0.001, β = 0.0051 and *p* < 0.001 and for the RD analysis, the overall model’s adjusted *r*^2^ = 0.37, *F* = 7.04, *p* < 0.001, β = −0.0193 and *p* < 0.001. Higher FA and lower RD of the splenium of corpus callosum predicted an increase in the changes in cognitive function. In the control group, there was no significant relationship between either FA (Fig. 2C) or RD (Fig. 2E) values in the right splenium of corpus callosum and changes in cognitive function.

## Discussion

The current study explored the role of baseline demographical characteristics, neuropsychological abilities and brain morphology on predicting the outcomes of memory training. The results indicate that older adults with lower years of education, thinner cortices of precuneus, cuneus and posterior cingulate gyrus and higher white matter anisotropy in splenium of corpus callosum predicted a higher cognitive improvement after MMT. Meanwhile, none of the baseline neuropsychological abilities predicted any cognitive improvement.

The participants’ educational level emerged as a significant predicting factor for the effects of MMT. Thus, asking one’s educational level could be an effective and easy way to predict the outcome of MMT before the training. Since the years of education is a multifaceted factor that reflects an individual’s amount of knowledge and cognitive ability, it is difficult to delineate a single possible explanation for this result. However, the theory of cognitive reserve suggests a plausible interpretation^24^. Cognitive reserve refers to the individual differences in the neural network underlying the performance of any task, which determines the coping ability and cognitive strategy against brain pathology. According to the theory, education is a crucial factor in determining the cognitive reserve^25^. Highly educated adults can cope with cognitive aging by utilizing many cognitive strategies based on their cognitive reserve, but those who are less educated cannot do so due to their lower cognitive reserve. Therefore, people receiving the MMT who are less educated can cope with cognitive aging better than before the training. We speculate that these training effects may be greater in less educated older participants because of their lesser experience in monitoring or controlling memory than those with a higher level of education.

So far, however, the predictive effect of years of education has been inconclusive. Belleville and colleagues showed that higher level of education was related to a larger enhancement in delayed list recall after cognitive intervention program in 47 participants (28 individuals with mild cognitive impairment and 17 normal controls)^9^. In the current study, only lower level of education was associated with cognitive improvement. These discrepancies can be attributed to the difference in the factors included in the study. The outcome of intervention can be affected by several factors such as the amount of cognitive impairment at the entry of intervention or the contents of intervention^26^. The characteristics of the MMT, which emphasize memory strategies and memory monitoring abilities, may explain the observed differences in outcome predictors compared with other studies. Belleville and colleagues^9^ trained individuals with mild cognitive impairments and healthy older adults to divided attention and mnemonic strategies (i.e., method of loci, PQRST; preview, question, read, state, test). The training was focused on the improvement of episodic memory (e.g., face-name association). Since the MMT of the current study focused on improving meta-memory abilities based on the knowledge of the memory and mnemonic strategies, the contents of intervention for improving higher-order cognitive functions (i.e., metamemory) were different from those of the previous one. It is expected that participants with less experience in self-awareness or control of their memory would benefit more from the MMT. This results concord with brain imaging results, which show that thinner cuneus (BA 17), precuneus (BA 7) and posterior cingulate cortex (BA 31) predict higher levels of cognitive improvement. These regions are related to cognitive reserve^27^ and self-awareness of memory^28,29^. Cuneus and precuneus, located within occipital and superior parietal lobes, are involved in strategic learning^30^. Precuneus and posterior cingulate cortex are also important for strategical approaches in memory^31^. The intertwined relationship between reduced cortical thickness, lower educational level and improvements following MMT needs further investigation.

The participants recruited for this study had subjective memory complaints only, not mild cognitive impairment where higher education has been reported as a predictor of positive training effect^9^. While none of the participants were diagnosed as mild cognitive impairments, there has been a report that older adults with subjective cognitive complaints showed a brain atrophy pattern similar to that of mild cognitive impairment^32^. Future study with assessment of interaction effects between different groups (e.g., subjective versus mild cognitive impairment) and training outcomes are needed to understand the discrepancies between studies.

Thinner gray matter thickness of medial parietal cortex is significantly related to cognitive and memory improvement (results presented in the Supplementary Material) after the MMT. Since training or therapy can increase brain volume^33,34^ smaller gray matter thickness of these regions before training may reflect a larger potential for the individual to improve on. However, there has been opposite reports as well; for example, a larger baseline hippocampal volume was related to improvement in recall^11^. These inconclusive results indicate that there are other variables moderating the relationship between brain volume and treatment outcomes, such as white matter microstructure or education. We speculate that if, for instance, constituents of the white matter microstructure, including axon density, axon diameter and myelination, are relatively optimal before the training begins, even an individual with relatively reduced cortical thickness could benefit much from training.

Individual differences in white matter anisotropy has been shown useful in predicting treatment outcome in subjects with substance abuse^35,36^. In this study, higher white matter anisotropy of splenium of corpus callosum positively predicted cognitive enhancement and those of right and left crura of fornix positively predicted memory enhancement (results were presented in the supplementary), in the training group. These results show that different microstructure features in hippocampus and other memory networks play a role in memory consolidation is necessary for cognitive and memory improvements. The reduction of the anisotropy of splenium of corpus callosum, which is hypothetically induced by a variety of factors such as membrane permeability, fiber density, or myelination breakdown, were observed at the early stage of Alzheimer’s disease^37^. Higher anisotropy of splenium of corpus callosum is linked with successful cognitive aging^38^ and better neuropsychological performances^39,40^. Therefore, white matter anisotropy of splenium of corpus callosum is a good predictor of the cognitive improvement after the MMT. Furthermore, fornix is the passage through which information is transferred between medial temporal lobe and medial diencephalon^41^ and thus is important for memory performances^42^. One study has found that bilateral injury of the fornix crura can trigger neural reorganization of the brain tract including splenium of corpus callosum in patients^43^. Since the crus of fornix has a crucial role in memory performance and neural reorganization, its microstructure at the entry of the training would be able to predict memory enhancement.

There are several limitations and suggestions for further studies. First, the sample size of the control group was relatively small (*n* = 10). This may lead to lower statistical power, reducing the chance of detecting a true effect in the control group. Even though the main purpose of the current study is to find the predicting factors of training effect within the training group, further study would require a larger sample size in the control group as well. Second, there could be other important predicting factors that were not observed in this study. For instance, the participants’ personalities or motivation for participating in memory training could have effects on the training outcomes. Future study should be performed with the participants’ motivational factors, such as self-regulation, interest or value, in consideration. Third, it is possible that the predictors found in this study were only specific to the MMT. Thus, further studies should be performed to generalize the predictors in other memory training programs.

Despite these limitations, the current study addresses an important niche in the brain structure and training literature. Data examining neuroanatomical characteristics have found that brain morphology can predict the performance of cognitive tasks^15^. However, few studies have examined the evidence regarding who will benefit from such memory trainings. The data presented in this report provides an initial point of reference indicating that the initial educational level and brain morphology could be promising variables for predicting cognitive enhancement after MMT.

## Method

### Participants

A sample of 49 older adults aged over 55 years with subjective memory complaints was recruited from nine community centers for elders. Among them, 17 (34.7%) were male. The sample was drawn from a larger original study which involved 112 individuals in training group and 89 in control group, currently under submission (Lee *et al*. (2016)), finding the effects of cognitive improvement and brain changes after MMT (Lee, J. *et al.*, under review). Among the 49 participants, 39 were selected from a group of 112 participants attending the MMT and other 10 were selected from a group of 89 controls who were not. The 49 people were randomly selected from those who expressed interest in brain imaging.

Exclusion criteria were: age <55 years; dementia; alcohol or substance abuse; head trauma; brain lesion on MRI; severe medical illness; severe neurologic or psychiatric disorders; currently taking medications likely to affect cognitive functioning; visual or hearing difficulties that interfere with the test tasking procedure, or; motor impairment that can affect test scores. Two geriatric psychiatrists screened the participants for dementia and other disorders based on the criteria of the fourth edition of the Diagnostic and Statistical Manual of Mental Disorders (DSM-IV)^44^. Mild cognitive impairment patients were also excluded according to Peterson’s criteria^45^.

The experimental protocol was approved by the Institutional Review Board of Seoul National University of Medicine. All participants gave written informed consent and all methods were performed in accordance with the relevant guidelines and regulations, as approved by the Institutional Review Board.

### Multi-strategic Memory Training (MMT)

The metamemory training (MMT) program was developed by Youn and colleagues (2011) based on the concept of metamemory for usage by healthy Korean older adults^13^. MMT consists of 10 sessions of memory training, once a week and each at a length of 1 hour and 30 minutes for a total of 15 hours. Each session is divided into three parts and each part takes 30 minutes. Part 1 concerns the delivery of knowledge regarding memory based on the metamemory concept; Part 2 involves training of metamemory-based memory strategies; Part 3 includes training of memory tasks in real-life situations. Metamemory is a type of metacognition and is composed of the knowledge of one’s own memory strategies and the abilities involved in monitoring and controlling one’s own memory. MMT lets the participant learn how memory strategies operate within the brain and how to cope with forgetting memories as one ages and improve on the ability to apply memory strategies in lifetime situations. MMT also includes meta-monitoring by letting the participants monitor their own level of memory and utilization of memory strategies during daily lives and share their stories with one another every session.

In the first session, a general introduction of the program is made and the participants share their experiences of forgetting with each other. The instructor gives a general explanation of the MMT and educates the participants on how to more efficiently utilize memory strategies and cognitive training in daily lives. Lastly, as a means of self-introduction, the participants of the program talk about their past experiences of forgetting. The participants discuss on the incidents in which they were discommoded by deterioration in memory function and how they had managed the situations. Through this discussion, each participant can recognize that problems such as inconvenience, decline in self-esteem, negative thoughts about aging, depressive mood and anxiety, discord within family, etc. are not experienced only by oneself.

In the second and third sessions, the instructor explains how one forgets with aging. The instructor motivates the participants to be more involved in the training process by explaining that forgetting increases over age because while the working memory span decreases very slowly and gradually over a long period of time, we tend to encode and retrieve information at the same speed despite being older. Then, the instructor educates the participants how to cope with forgetting by utilizing metamemory. Through a metamemory-based strategy training, the participants develop skills to input information in accordance with their narrower working memory span and withdraw memory more slowly. This training is constructed so to enhance the participants’ memory strategies. Moreover, through meta-monitoring, the participants can check their current states by utilizing their enhanced meta-cognition and share them with one another through discussion. Lastly, the participants are given assignments to employ the coping methods for forgetting in their daily lives. The coping strategy and training assignment for the second session is to speak out loud what they are currently doing; for the third session it is to illustrate a mental image of their actions.

In sessions number 4 to 9, the instructor begins Part 1 by giving an explanation about either the structure of memory (4^th^ and 5^th^ sessions); attention (6^th^ session); the brain (7^th^ session); the environment (8^th^ session), or; perception (9^th^ session). Then, in Part 2, the participants are trained of the specific steps of memory strategies. The instructor informs the participants on memory strategies such as story-making, imagery, organization and method of loci *via* a variety of visual media and educational materials. In Part 3, the participants discuss on how the memory strategies learned in each session can be applied in real-life situations, and are encouraged to actually put the strategies to use through assignments.

The tenth session is a wrap-up session; essential points from the previous trainings are presented in a summarized form and the participants share their thoughts on the training and their experiences on memory and forgetting. Through this sharing process, the instructor encourages the participants to continuously train and utilize the memory strategies in daily lives.

### Neuropsychological Measures

#### Mini Mental State Examination (MMSE)

The MMSE is a neurocognitive test designed to screen cognitive impairment^46^ with the score ranging from 0 to 30. Higher score indicates better cognition. The Korean version of MMSE consists of orientation (10 points), short-term memory registration and recall (6 points), attention (5 points), naming (2 points), following verbal commands (4 points), judgment (2 points), and copying a double pentagon (1 point). It has been validated for use among Korean elders^47^.

#### Elderly Memory Disorder Scale (EMS)

EMS was used to assess the participants’ cognitive function^48^. It was developed and standardized for the Korean elderly population. It consists of tests that evaluate verbal memory (Elderly Verbal Learning Test) and nonverbal memory (Simple Rey Figure Test), executive function (Digit Span Test; DST, Spatial Span Test; SST) and language ability (Categorical Fluency Test; CFT, short version of Boston Naming Test; sBNT).

Elderly Verbal Learning Test was developed to examine verbal memory. Participants were given 9 words from 3 categories and then asked to recall the word, once immediately and once after a delay of 20 minutes. The performances of the two stages were scored for a total score of 9. Simple Rey Figure Test, a simplified version of Rey-Osterrieth Complex Figure Test (RCFT), was used to investigate nonverbal memory. Geometric figures were presented to the participants, who were told to copy the figure (copying stage) and reproduce it from memory (recall stage). The results were scored from 0 to 16. DST and SST were used to evaluate executive function and attention. In the DST, participants were told a series of numbers and asked to repeat the list either forwards or backwards. In the SST, 10 regular hexahedrons were located in a board and tapped in a certain sequence; the participants were told to mimic the sequence either forwards or backwards. The results were recorded for a total score of 14. CFT and sBNT were used to examine language ability. In the CFT, participants asked to name as many animals as possible within a minute. In the sBNT, 15 pictures were presented and participants were asked to name each presented stimulus. The result for each test was scored from 0 to 15.

### Statistical analysis

#### Computation of changes in cognitive function

Since the MMT training is a multistrategic training based on the metamemory concept, the outcome measures were not only focused on memory but also focused on the transfer effects on general cognitive functions. Therefore, the changes in cognitive function were computed.

To investigate the relationships between the initial individual differences (i.e., demographic and neuropsychological characteristics, neuroanatomical structures) and cognitive ability enhancement after training, the latter was calculated as the difference in cognitive performances between baseline and follow-up study. The difference was calculated in three steps. First, all 10 outcome variables in EMS (i.e., word list and delayed free recall in Elderly Verbal Learning Test, Simple Rey Figure Test copy and delayed free recall, DST forward and backward, SST forward and backward, CFT and sBNT) were standardized to the respective z-scores of training and control groups. Two sets of standardized variables, pre- and post-training, were computed separately in this process. That is, each participant had a total of twenty standardized dependent variables (i.e., the 10 outcome variables in EMS from the pre- and post-training assessments). Second, the means of the 10 variables from either pre- or post-sets were calculated in each group. The values were termed as either pre-score or post-score. Through this process, two mean values were assigned to each participant. Third, post-score minus pre-score was computed, producing one value for each participant. This subtracted value, named as relative performance change in cognitive function, indicates the extent of relative improvement of cognitive ability after the training in the training group. The same value for the control group indicates cognitive score changes between first and second assessments without any training.

### Behavioral data analysis

PASW 18.0 for WINDOWS was used for data analyses. A *t*-test and chi-square tests were run across the demographic variables (age, education, MMSE and gender) in order to verify homogeneity between the training and control groups. A repeated-measures analysis of variance (rmANOVA) was conducted to examine the effects of the metamemory training on neuropsychological tests. Analyses were performed with either the training or control group as a between-subjects variable and the neuropsychological testing performances on the first and second follow-up phases as the within-subjects factor.

Stepwise multiple linear-regression, demographic variables (i.e., age, gender, years of education) and all 10 measures of EMS (i.e., word list and delayed free recall in Elderly Verbal Learning Test, Simple Rey Figure Test copy and delayed free recall, DST forward and backward, SST forward and backward, CFT and sBNT) were used as possible independent variables and pre-training set on changes in cognitive function was used as dependent variable. A *p* value of < 0.05 derived from the two-tailed test were considered statistically significant.

### Brain imaging acquisition and processing

#### MRI data acquisition and processing for cortical thickness

T1-weighted MR images were acquired using the 3.0-Tesla MRI scanner (Philips, Achieva) with the following imaging parameters: repetition time (TR), 9.9 ms; echo time (TE), 4.6 ms; flip angle, 8°; field of view (FOV) of 220 mm and matrix size of 220 × 220 pixels; slice thickness, 1 mm; and voxel size of 1 × 1 × 1 mm.

Structural MRI data were automatically processed by the CIVET pipeline to measure the cortical thickness^49^. It is well-validated and has been extensively described in other studies dealing with aging and atrophied brain^50–55^. Detailed steps of image processing were described in Zijdenbos *et al*.^49^. In brief, the steps included: correction for intensity nonuniformity^56^; normalization to the MNI 152 template^57^; removal of non-brain tissues^58^; tissue classification of white matter, gray matter, cerebrospinal fluid and background^59^; and surface extraction of the inner and outer cortices^60,61^. A surface model for each hemisphere consisted of 40,962 vertices. The surfaces were transformed back into the native space and the cortical thickness was measured as the Euclidean distance between the linked vertices of the inner and outer surfaces^51^. To compare the thicknesses of corresponding vertices among the subjects, the vertices of the surfaces were spatially registered onto a MNI 152 surface template using sphere-to-sphere warping algorithm^62,63^. The cortical thicknesses were blurred using a smoothing kernel with a full-width half-maximum of 20 mm to increase the signal-to-noise ratio and statistical power.

To evaluate the relationship between baseline cortical thickness (i.e. pre-MRI time point) and changes in cognitive function, a linear regression analysis was applied on a vertex-by-vertex basis with age, sex and intracranial volume (ICV) as covariates using SurfStat toolbox (http://www.math.mcgill.ca/keith/surfstat/) for Matlab (R2014a, The MathWorks, Inc.). ICV is defined as the total volume of gray matter, white matter and cerebrospinal fluid. Since the ICV reflect an individual’s neural reserve, we used ICV as a control variable. To remove the variance accounted for ICV, it was calculated by measuring the volume of voxels within the brain mask that was generated using the FMRIB Software Library bet algorithm^58^. Statistical significance was assessed at a corrected *p* value of <0.05 by RFT to correct for the multiple comparisons^64^ with a cluster area of >100 mm^2^. We neglected small areas of cluster (<100 mm^2^) since it is difficult to ascribe specific functional significance to areas of such small sizes in the cerebral cortex. Furthermore, multiple regression analysis was performed with average cortical thickness extracted from each cluster with age, gender and ICV as independent variables and changes in cognitive function as the dependent variable.

### DTI data acquisition and processing

Diffusion-weighted images were acquired using the 3.0-Tesla MRI scanner (Philips, Achieva). For DTI, a single-shot twice-refocused spin echo planar imaging (EPI) pulse sequence with 32 diffusion-sensitized gradient directions was utilized with the following imaging parameters: b-value, 1000 s/mm^2^; repetition time (TR), 7259 ms; echo time (TE), 68 ms; flip angle, 90°; FOV of 220 mm and matrix size of 128 × 128 pixels; slice thickness, 2 mm; and voxel size of 1.53 × 1.53 × 2 mm. The DTI data were processed using FMRIB’s Software Library (http://www.fmrib.ox.ac.uk/fsl). Motion artifacts and eddy current distortions were corrected by normalizing each diffusion-weighted volume to the non-diffusion-weighted volume (b_0_) using affine registration method in the FMRIB’s Linear Image Registration Tool (FLIRT). Diffusion tensor matrices from the sets of diffusion-weighted images were generated using a general linear fitting algorithm. Subsequently FA, RD and AD were calculated for every voxel according to standard methods.

The FA and the MD maps of DTI preprocessing results were used in TBSS analysis^65^. All FA images were aligned onto the standard FMRIB58 FA template provided in the FSL software using a nonlinear registration algorithm implemented in the TBSS package. The FA images aligned on the FMRIB58 FA template were averaged to create a skeletonized mean FA image. Each subject’s aligned FA images were projected onto the skeleton by filling the skeleton with the highest FA values from the nearest relevant center of fiber tracts. A threshold FA value of 0.2 was chosen to exclude voxels of adjacent gray matter or cerebrospinal fluid. For analysis, the images were also processed by applying the FA non-linear registration and projecting them onto the skeleton using identical projection methods to those inferred from the original FA data. Then, voxel-wise statistics across subjects on the skeleton-space FA images were carried out.

Furthermore, to identify the brain regions correlated with the changes in cognitive function, a multiple regression analysis was performed with the average FA values in different brain regions, with age and gender as independent variables and changes in cognitive function as the dependent variable. The Johns Hopkins University (JHU)-ICBM-DTI-81 atlas within the FSL program was used to guide the placement of the ROIs (http://www.fmrib.ox.ac.uk/fsl/fslview/atlas.html). The results were considered significant at a corrected FDR-corrected *p* < 0.05 to control for the multiple comparisons.

### Procedures

Older adults with subjective memory complaints who have visited the SMG-SNU Boramae Medical Center of South Korea have volunteered to participate in the MMT.

Randomization procedure was carried out to categorized participants into the training and control groups. A random digit was generated for each participant according to the table of random numbers. If the random digit was an even number, the participant was assigned to the control group; if it was an odd number, the participant was assigned to the training group.

Demographic variables (age, gender and years of education) and neuropsychological measures (i.e., MMSE, EMS) were evaluated at the entry of the training. Two blind, trained clinical neuropsychologists conducted the neuropsychological assessments. Post-training neuropsychological evaluations *via* EMS were conducted within three-month after finishing the training. Program participation was free of charge and there was no financial reward for participation. Among the participants, 49 were randomly selected to test for the changes in the brain structure. Magnetic resonance imaging was conducted twice, prior to the training (pre-training evaluation) and 6 months after the training (post-training evaluation).

## Electronic supplementary material

Supplementary materials
