# Supplementary material for: Neural predictors of cognitive improvement by multi-strategic memory training based on metamemory in older adults with subjective memory complaints
Source: Sci Rep. 2018 Jan 18;8:1095. doi: 10.1038/s41598-018-19390-2 (PMC5773558; doi:10.1038/s41598-018-19390-2)
Supplement: Supplementary file 1 — Supplementary materials [file 41598_2018_19390_MOESM1_ESM.pdf]

Neural predictors of cognitive improvement by multi-strategic memory training based on metamemory in older adults with subjective memory complaints

(SHORT TITLE: Neural predictors of cognitive improvement)

Soowon Park[1], Seung-Ho Ryu[2], Yongjoon Yoo[3], Jin-Ju Yang[4], Hunki Kwon[4, 5], Jung-Hae Youn[6], Jong-Min Lee[4], Seong-Jin Cho[7], Jun-Young Lee[8*]

[1]Department of Education, Sejong University, Seoul, Republic of Korea

[2]Department of Psychiatry, School of Medicine, Konkuk University, Konkuk University Medical Center, Seoul, Republic of Korea

[3]Seoul National University College of Medicine, Seoul, Republic of Korea

[4]Department of Biomedical Engineering, Hanyang University, Seoul, South Korea

[5]Department of Neurology, Yale University School of Medicine, New Haven, CT, USA

[6]Graduate School of Clinical & Counseling Psychology, CHA University, Gyeonggi-Do, Republic of Korea

[7]Department of Psychiatry, Gachon University of Medicine and Science, Incheon, Republic of Korea.

[8]Department of Psychiatry, Seoul National University & SMG-SNU Boramae Medical Center, Seoul, Republic of Korea.

*Corresponding author:

Jun-Young Lee, SMG-SNU Boramae Medical Center, 20 Boramae-Ro 5-Gil, Seoul 07061, Republic of Korea.

Email: benji@snu.ac.kr

Supplementary Results

Results for the changes in memory performance

Changes in memory performance

The standardized memory assessment scores (i.e., z-scores) pre- and post-training in the training group are presented in Supplementary Figure 1 (right). The same data from the control group are presented in Supplementary Figure 2 (right). The change in each memory ability was computed by subtracting pre-z-score from post-z-score, the results indicating the slope of each line. Median changes in standardized memory scores was 0.069 in the training group and 0.069 in the control group.

Cortical thickness predictors for changes in memory performance

In the training group, significant relationships between the cortical thickness and changes in memory performance [RFT corrected; $p < 0.05$; overall model's adjusted $r^2 = .29$; $F = 4.87$; $p < .005$; cluster area = 202.41 mm^2] were found in the area consisting of right precuneus (BA 7) and posterior cingulate gyrus (BA 31) [$\beta = -1.72$; $t = -4.09$; peak MNI coordinate (x, y, z) = (6.15, -56.48, 12.44)].

Supplementary figure 3A indicates the brain regions showing significant correlations with the changes in memory function in the training group. The regression graph of the averaged cortical thickness on cluster at the entry of the training versus changes in memory performance is presented on Supplementary figure 3B. In the control group, there was no significant relationship between cortical thickness and changes in memory function.

White matter structure for predicting changes in memory performance

In the training group, DTI analysis showed that the changes in memory performance were positively correlated with the averaged FA values in the crura of fornix after controlling for age and gender (Figure 3). For the FA analysis of right crus of fornix, the overall model's adjusted $r^2 = .43$, $F = 10.65$, $p < .001$, $\beta = 0.0258$ and $p < .001$ and for the FA analysis of left crus of fornix, the overall model's adjusted $r^2 = .23$, $F = 4.63$, $p < .005$, $\beta = 0.0506$ and $p < .001$. Higher FA of the crus of fornix predicted an increase in the changes in memory performance. In the control group, there was no significant relationship between the FA values in the crus of fornix and the changes in memory performance.

Method

Computation of changes in memory performance

Computation of changes in memory performance was done in the same way as the computation of changes in cognitive function, except that only memory assessments (i.e., word list and delayed free recall in Elderly Verbal Learning Test, Simple Rey Figure Test copy and delayed free recall) were included.

Supplementary figures

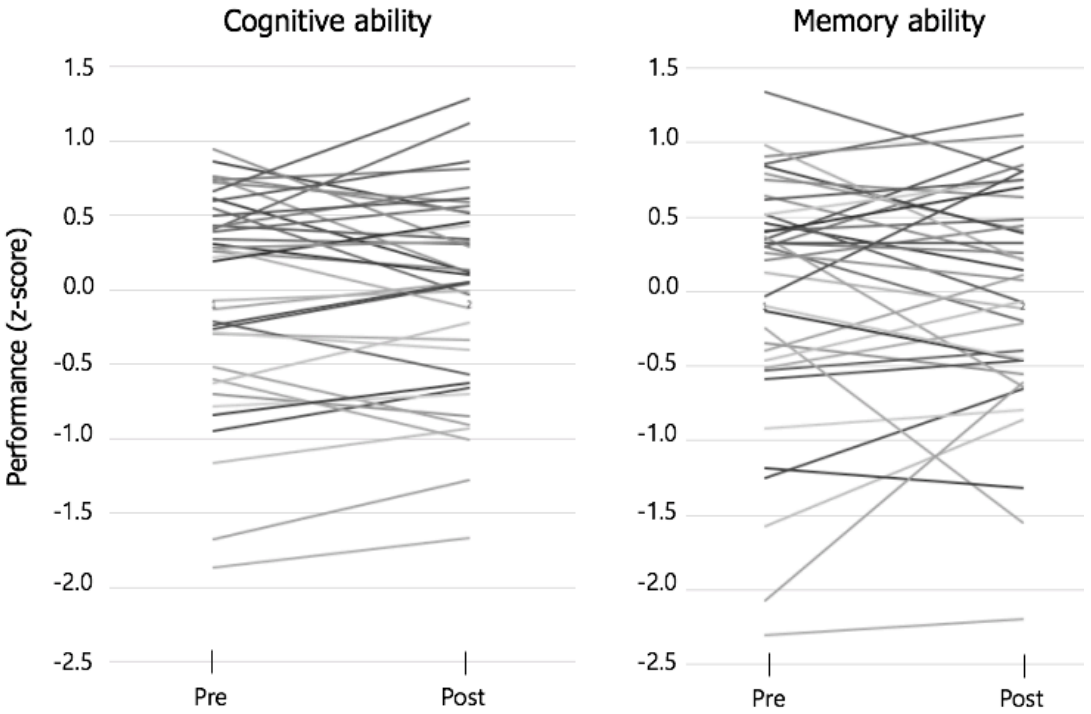

Supplementary figure 1. The standardized scores (i.e., z-scores) at the pre- and post-neuropsychological assessments in the training group (n = 39; left: cognitive performances; right: memory performances). The change in each cognitive ability or memory ability was computed by subtracting the pre-z-score from the post-z-score, indicating the slope of each line.

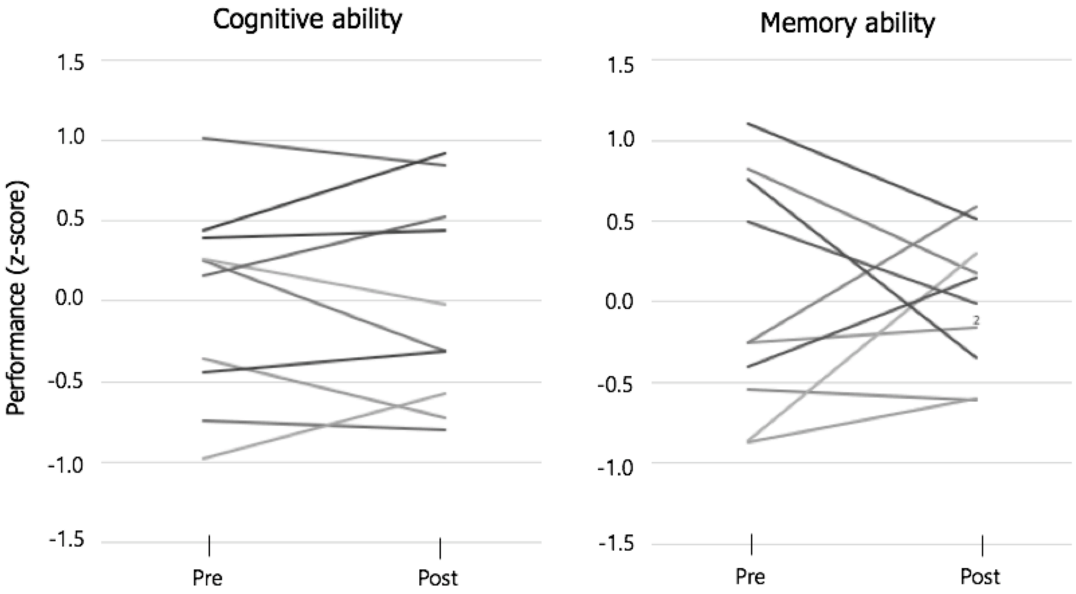

Supplementary figure 2. The standardized scores (i.e., z-scores) at the pre- and post-neuropsychological assessments in the control group (*n* = 10; left: cognitive performances; right: memory performances). The change in each cognitive ability or memory ability was computed by subtracting the pre-z-score from the post-z-score, indicating the slope of each line.

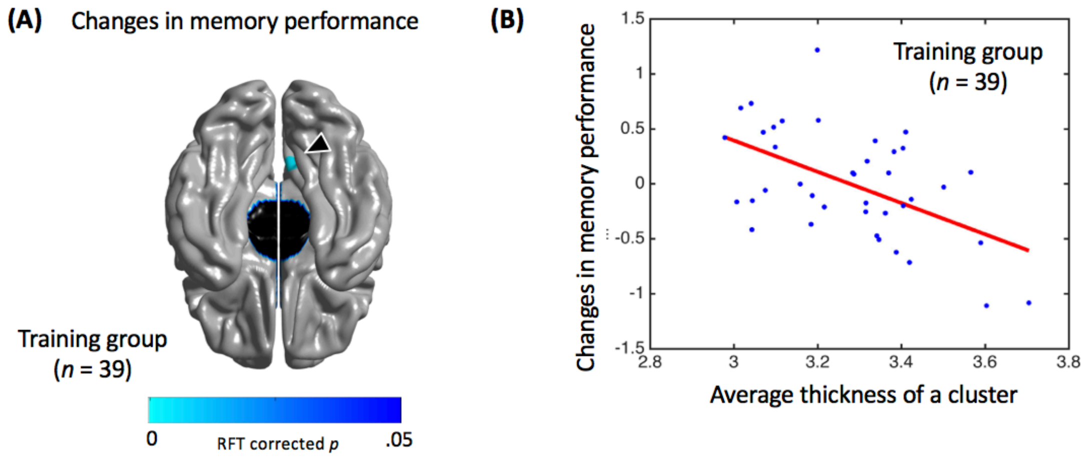

Supplementary figure 3. (A) Brain map showing the clusters with a significant correlation between cortical thickness and changes in memory performance at right precuneus (BA 7) and posterior cingulate gyrus (BA 31), which emerged when the P map indicated by the color was corrected for multiple comparison at a 0.05 threshold. The cluster is indicated by the triangle. (B) Regression graph of the averaged cortical thickness of clusters at the entry of the training according to changes in memory function (adjusted r^2 = .29; F = 4.87; p < .005; B = -1.72; p <.001).

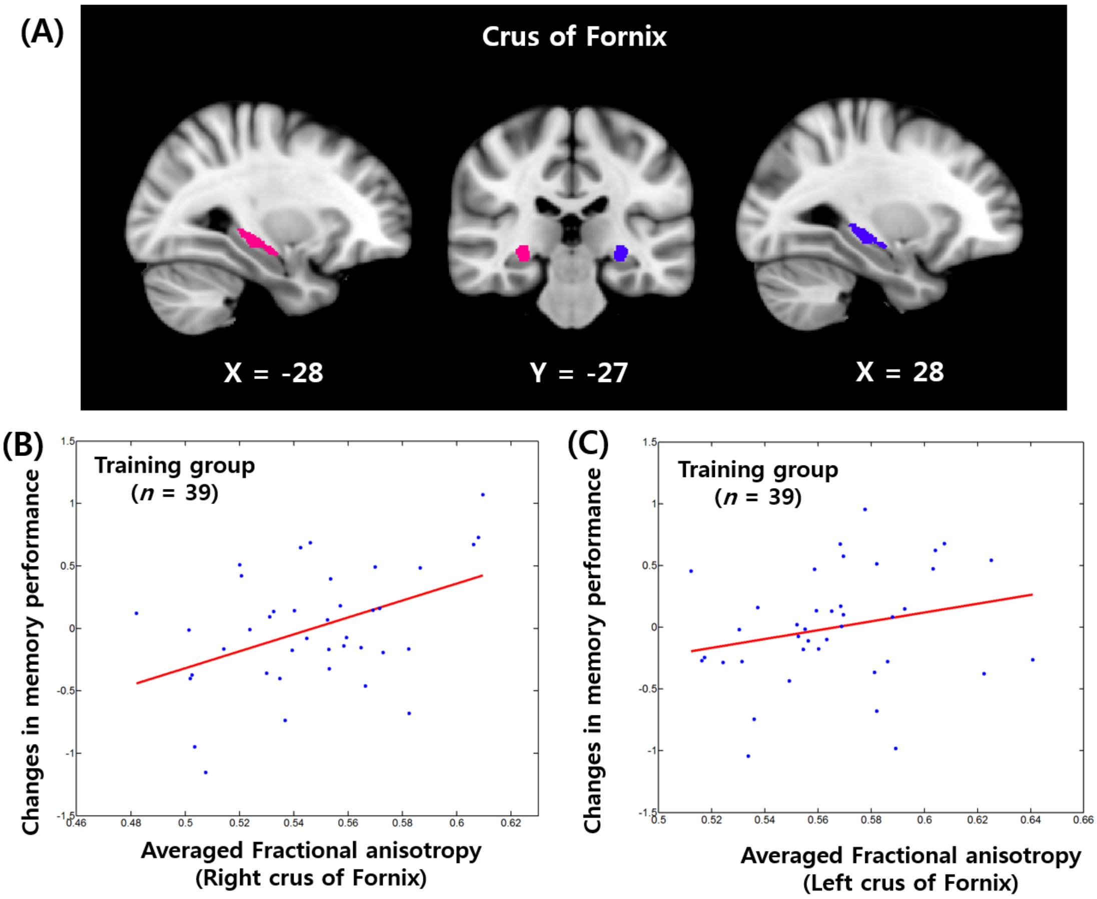

Supplementary figure 4. (A) The location of the right and left crura of fornix. (B) The relationship between the averaged fractional anisotropy and the changes in memory function (overall model's adjusted r^2 = .33; F = 8.68; p < .001; B= 5.61; p < .001) of the right crus of fornix. (C) The relationship between the averaged fractional anisotropy and the changes in memory function (overall model's adjusted r^2 = .35, F = 9.54, p < .001, β = -0.0001, p < .001) of the left crus of fornix. The results were significant at a corrected false discovery rate-corrected p < 0.05 to control for the multiple comparisons.
